# Creutzfeldt-Jakob Disease Presenting as Expressive Aphasia and Nonconvulsive Status Epilepticus 

**DOI:** 10.1155/2018/5053175

**Published:** 2018-02-14

**Authors:** Hafiz B. Mahboob, Kazi H. Kaokaf, Jeremy M. Gonda

**Affiliations:** ^1^University of Nevada School of Medicine, Reno, NV, USA; ^2^Renown Regional Medical Center, Reno, NV, USA

## Abstract

Creutzfeldt-Jakob disease (CJD), the most common form of human prion diseases, is a fatal condition with a mortality rate reaching 85% within one year of clinical presentation. CJD is characterized by rapidly progressive neurological deterioration in combination with typical electroencephalography (EEG) and magnetic resonance imaging (MRI) findings and positive cerebrospinal spinal fluid (CSF) analysis for 14-3-3 proteins. Unfortunately, CJD can have atypical clinical and radiological presentation in approximately 10% of cases, thus making the diagnosis often challenging. We report a rare clinical presentation of sporadic CJD (sCJD) with combination of both expressive aphasia and nonconvulsive status epilepticus. This patient presented with slurred speech, confusion, myoclonus, headaches, and vertigo and succumbed to his disease within ten weeks of initial onset of his symptoms. He had a normal initial diagnostic workup, but subsequent workup initiated due to persistent clinical deterioration revealed CJD with typical MRI, EEG, and CSF findings. Other causes of rapidly progressive dementia and encephalopathy were ruled out. Though a rare condition, we recommend consideration of CJD on patients with expressive aphasia, progressive unexplained neurocognitive decline, and refractory epileptiform activity seen on EEG. Frequent reimaging (MRI, video EEGs) and CSF examination might help diagnose this fatal condition earlier.

## 1. Introduction

We report a rare clinical presentation of sporadic CJD (sCJD) with combination of both expressive aphasia and NCSE. Isolated language problems and aphasia have been described in CJD before [1–8]; however, this combination is unique. This patient had an atypical clinical presentation with normal initial workup, but subsequent workup revealed CJD with typical EEG finding of spike-wave complexes (PSWCs) as well as hyperintensities in basal ganglia and cortical ribboning on MRI and positive CSF analysis for 14-3-3 proteins. Due to the extremely high mortality rate and often atypical clinical presentation and/or inconclusive initial workup, a high degree of suspicion and thus repeating workup might aid in early diagnose of this fatal condition.

## 2. Case Report

A 60-year-old male with a past medical history significant only for benign prostatic hyperplasia presented to our Emergency Department (ED) with chief complaints of gradual onset of progressively worsening speech difficulty (predominantly word finding with stuttering) and confusion (inability to recognize his family members).

His symptoms started four weeks priorly, beginning with constitutional symptoms of headache, fatigue, and vertigo. This slowly led to intermittent confusion, slurred speech, and intermittent spasms of his right upper and lower extremities. His spasms and weakness resulted in a fall from a tractor one week into the course of his symptoms fortunately without significant trauma nor loss of consciousness. His family gave additional potentially relevant information of a recent visit to Mexico where he stayed for four months before returning home. He was asymptomatic upon return to the USA, but symptoms started approximately four weeks later. Interestingly, while in Mexico, he worked in the cattle manure industry which he does locally as well. He was relatively healthy at baseline without any previous surgeries and no family history of diabetes, seizure, dementia, nor neurodegenerative conditions.

He initially visited an urgent care center with these complaints ten days prior to this hospital encounter. At that time, he was found to have a normal neurological examination, brain MRI, and carotid Doppler. He was discharged home with a working diagnosis of transient ischemic attack. His symptoms continued to worsen however, thus motivating him to present to the ED for further evaluation.

Upon arrival to the ED, he complained of photophobia, neck pain, and vertigo but was afebrile. His initial physical examination included vital signs: pulse 85 beats per minute, blood pressure 135/78 mmHg, temperature 98 F, and a respiratory rate of 18 per minute. Initial neurological examination was unremarkable except for persistent word finding difficulty. Initial lab work is summarized in table format (Table 1). A noncontrast head computed tomography (CT) scan was negative for any acute intracranial pathology. Initial CSF analysis was inconclusive for any acute infectious etiology although he had a mildly elevated protein level of 65 mg/dl (Table 2). He was admitted to the neurology unit and diagnosed with a complex migraine and treated with intravenous (IV) ketorolac, sumatriptan, and promethazine. A neurology consult was obtained and an EEG ordered.

**Figure 1:**
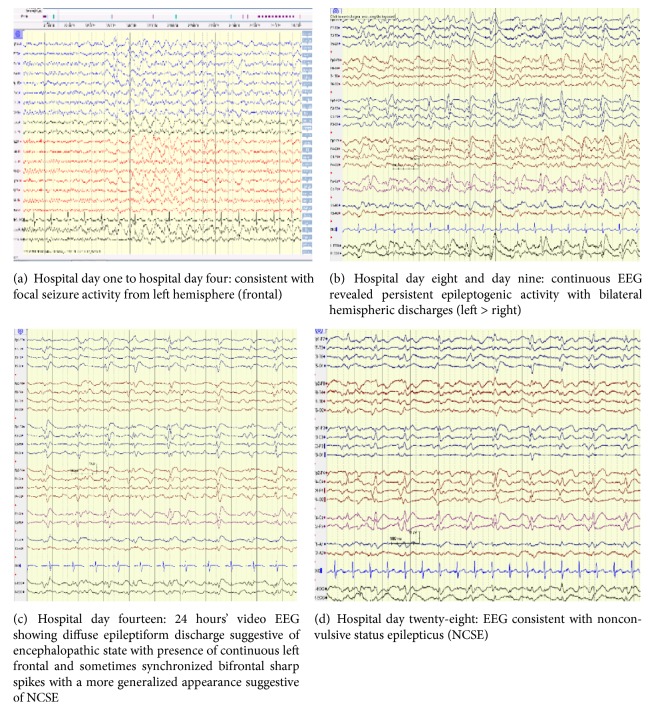
*EEG studies*.

Initial EEG on his second hospital day showed focal seizures emanating from the left frontal region (Figure 1(a)) and he was started on oral levetiracetam (loading dose of 1 gram followed by 750 mg PO twice daily thereafter). He started complaining of right upper extremity weakness and on repeat physical exam was found to have diminished deep tendon reflexes of his right upper extremity (RUE) with weakness in pronation and fine motor activity. On hospital day #3, he developed intermittent myoclonic jerking of his RUE with a fine, persistent tremor. At this point intermittent focal seizure activity was thought to be precipitated from his minor head trauma related to his fall. Differential diagnoses, though less likely, included an infectious etiology which was excluded with negative microbiology, a stroke despite a normal MRI, or other common metabolic causes including electrolyte abnormalities, ammonia, toxins, and liver and kidney dysfunction. Medications were also considered which could potentially lower seizure threshold, and tramadol and diphenhydramine were discontinued.

**Figure 2:**
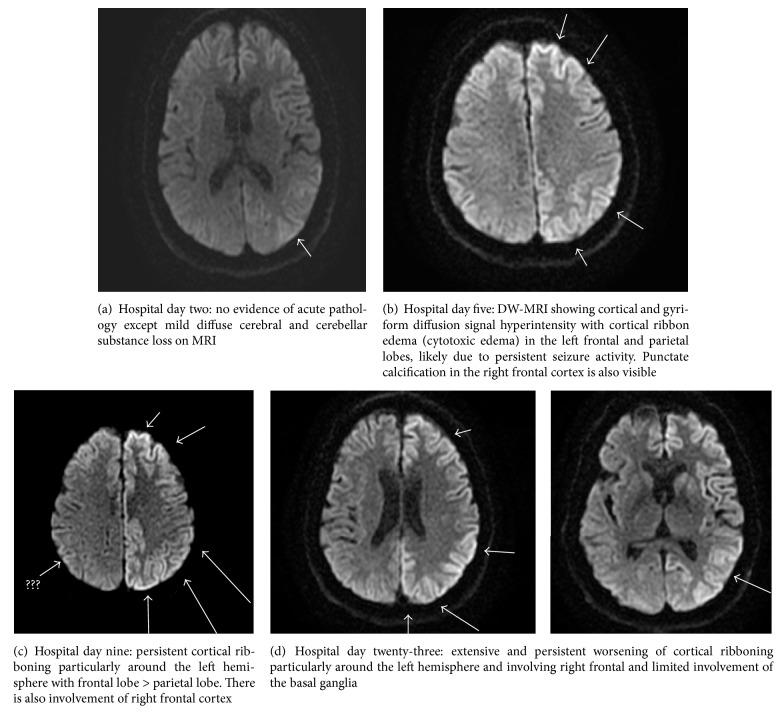
*MRIs*.

Over the next few days he gradually became drowsier and confused with worsening of his expressive aphasia and development of cerebellar dysfunction on exam. His dose of levetiracetam was increased to 1 gm twice daily. Initial MRI of brain during this hospital encounter was performed which did not show any acute intracranial lesion except mild cerebral and cerebellar substance loss (Figure 2(a)).

His cognition and right upper extremity shaking/tremor rapidly worsened and, on hospital day #4, EEG was significant for persistent focal seizures in left hemisphere despite being on levetiracetam (Figure 1(a)). He was started on lacosamide 200 mg twice daily in addition to the ongoing levetiracetam increased now to 1 gm three times daily. Continuous EEG was started at this point to closer monitor effectiveness of therapies. A repeat MRI brain with contrast on hospital day #5 showed developing cytotoxic edema in the left frontal and parietal lobe with punctate calcified lesions in right cortex (Figure 2(b)) which was considered likely due to persistent seizure activity. Given the initial CSF result showing elevated proteins (65 mg/dl), ongoing myoclonus, and the newly developed vasogenic edema on MRI, the decision was made by neurology at this point, to start a 3-day course of pulse dose steroids (solumedrol 1 gm IV/daily) for possible autoimmune encephalitis (AE) while awaiting the finalized autoimmune workup [9, 10].

Unfortunately, despite the increasing doses of antiepileptics and the high dose steroids, the patient continued to decline neurologically. Continuous EEG revealed persistent epileptogenic activity with bilateral hemispheric discharges (left > right) (Figure 1(b)). Valproic acid (500 mg orally three times daily) was added to his regimen and lacosamide increased to 200 mg three times daily. His initial CSF (collected on the first hospital day) was sent for oligoclonal bands and infectious encephalopathies which all eventually came back negative (Table 2). Acyclovir was started empirically for possible viral encephalitis but subsequently discontinued two days later when CSF resulted negative.

During the next few days (hospital days nine to eleven) he remained globally aphasic with RUE flaccidity. He would awaken but was unable to follow commands. A repeat MRI showed persistent ribboning in the left hemispheric region (Figure 2(c)) and EEG (Figure 1(c)) showed “diffuse epileptiform discharge suggestive of encephalopathic state with presence of continuous left frontal and sometimes synchronized bifrontal sharps spikes with a more generalized appearance, which was concerning for nonconvulsive status epilepticus.” Perampanel was added to his antiseizure regimen at dose of 4 mg twice daily. Efficacy of perampanel has been established as an adjunct treatment for partial-onset seizures with or without secondary generalization and primary generalized tonic-clonic seizures in idiopathic generalized epilepsy as well as for treatment of refractory seizures. This patient was having refractory seizures; therefore, this medication was added [11].

At this point CJD was considered among other possible etiologies such as paraneoplastic encephalitis, meningeal carcinomatous, infectious cerebritis, and primary CNS angiitis given his continued deterioration and refractory status epilepticus. Computed tomography angiogram (CTA) excluded the primary central nervous system (CNS) angiitis. A repeat lumbar puncture was performed with additional CSF tests ordered including Epstein-Barr virus (EBV), acid fast bacilli (AFB), fungal culture, Zika virus, 14-3-3 protein, neurocysticercosis antibodies, and an autoimmune and paraneoplastic panel.

Eventually phenobarbital (16.2 mg twice daily) was started yet he continued to deteriorate remaining aphasic with flaccid paralysis in RUE and lost his ability to protect his airway requiring intubation on hospital day #11. His EEG remained without any significant improvement. On hospital day #13, while awaiting results from his repeat CSF analysis, neurology felt it was prudent to again trial high dose steroids (solumedrol 1 gm IV daily) for potential AE which continued for the next 5 days without obvious efficacy.

He remained in a persistent coma at this point and lost reflexes to even deep painful stimuli while cEEG showed continuous episodic sharp wave from left frontal and synchronized bifrontal discharge was obvious on lowering his sedation (Figure 1(c)). Seizures were refractory to extensive antiseizure medication regimen including keppra 1500 mg twice daily, lacosamide 200 mg three times daily, perampanel 4 mg twice daily, phenobarbital 120 mg twice daily, and valproic acid 1 gm twice daily.

On hospital day #23, his course was complicated by development of ventilator associated pneumonia, for which he was started on vancomycin and cefepime. Cultures from bronchoalveolar lavage were negative.

His neurological condition did not improve with EEG persistently showing frequent generalized epileptiform discharges (Figure 1(d)). Repeat MRI brain (Figure 2(d)) showed extensive and persistent worse cortical ribboning particularly around the left hemisphere, and involving right frontal, and limited involvement of the basal ganglia without any acute infarct on diffusion weighted imaging (DWI) (suggestive of cytotoxic edema from prolonged seizure activity and early CJD). Tissue diagnosis from brain biopsy was discussed but, given hospital policy and limitations, deemed not possible.

On hospital day #26, his CSF finally resulted from the national laboratory positive for 14-3-3 protein, tau protein, and Real-Time Quaking-Induced Conversion (RT-QuIC) proteins, confirming prion disease. Before giving his family the news of the results, and for prognostication purposes, his sedation was held for several hours. On physical exam, he lacked any withdrawal response to painful stimuli but maintained cough and gag reflexes with a minimal, sluggish pupillary reflex. EEG (Figure 1(d)) at that time showed a worsening seizure activity pattern which initially started as focal left frontal lobe diffuse spike-wave complexes but now had progressed into generalized epileptiform discharges with development of nonconvulsive status epilepticus (NCSE).

A family conference was held discussing the patient's terminal diagnosis and a decision was made to transition the patient to comfort care with compassionate extubation. He died shortly after extubation with family at bedside. The county health department was involved in his case and investigations were performed at the local manure plant to test for potential cow involvement, usually seen in variant (vCJD), which was unremarkable. However, our patient's age of disease onset, typical EEG (PSWC), absent pulvinar sign on MRI as well as lack of past medical/surgical and family history was not consistent with vCJD. Thus, he was diagnosed with probable sCJD. His remains were cremated, and his ashes taken to Mexico by family preventing final genetic testing to be performed.

## 3. Discussion

CJD is the most commonly seen form of prion diseases in humans [1]. This is a fatal neurodegenerative disease which typically results in subacute and progressive deterioration in cognitive, behavioral, and motor function over a period of weeks to months [12–15]. Typical clinical presentation also includes startle myoclonus, cerebellar, pyramidal, extrapyramidal, behavioral, and visual defects with a characteristic periodic sharp complex on the EEG [16, 17]. Other neurocognitive disorders including Alzheimer's disease, Lewy body dementia, vascular dementia, and frontotemporal dementia tend to have slower course and gradual cognitive decline [18].

Based on its etiology, CJD is divided into sporadic (most common), variant (vCJD), iatrogenic, and familial forms [14, 19]. Genetic analysis of prion protein gene (PRNP) can help to identify different forms of CJD and to subclassify them based on molecular phenotype [20, 21].

Sporadic form is seen in older age and previous diagnosis of psychosis, multiple surgeries, and living in the farm (garden or animal farm) for more than 10 years are associated risk factors [22]. Our patient had worked in a cow manure plant although investigations performed at the local manure plant to test for potential cow involvement were unremarkable. Variant CJD is seen in younger age and represents bovine-to-human transmission. It manifests with early sensory disturbances and psychiatric symptoms rather than cognitive decline [23]. EEG usually do not show PSWCs and a slow wave pattern is predominant [20, 24]. Symmetrical hyperintensity in the pulvinar nuclei of the thalamus termed as “pulvinar” sign is 90% specific for vCJD [25, 26]. Our patient in terms of clinical presentation (age, personal and family history, EEG, and MRI findings) falls under sCJD.

Although the diagnosis may be straightforward in older adults who present with the classic clinical and radiological presentation, the diagnosis becomes challenging if the initial presentation is atypical both clinically and in terms of imaging [27].

CJD have presented with a variety of atypical clinical syndromes including but not limited to amyopathy, deafness, and cataracts [28–30] and often with nonspecific constitutional signs and symptoms such as dizziness [12]. Our patient also did have a work-related fall and difficulty waking but he did not have evidence of amyopathy (absence of fasciculation and areflexia). His right upper extremity weakness was likely related to involvement of left frontal cortex with seizure activity resulting in “postictal” weakness. He did not have an isolated period of constitutional symptoms before cognitive decline as some other patients did [12]. He did not have deafness or cataracts. He did have vertigo, which is hard to differentiate if being of central or of peripheral origin.

CJD is a rare but an important stroke mimic making it challenging to differentiate between the two, as CJD develops mostly in elderly population who usually also have risk factors for stroke [27]. Our patient had no evidence of stroke.

Seizures have been reported in up to 15% of patients with CJD during the disease course [1]. However, seizures are reported as initial manifestation of the CJD disease only in 3% of cases [31]. Status epilepticus is reported in less than 15% of patients of sCJD [14]. Our patient's EEG identified focal seizure activity early during the hospitalization which then progressed to NCSE as patient deteriorated.

Akinetic mutism is a known entity in patients in the final stages of CJD [32]. In contrast to akinetic mutism (again seen in the late stages), patients can have aphasia as a manifestation of CJD [3–8]. Aphasia at disease onset, however, is much less common [2]. This patient is a rare clinical presentation of sporadic CJD (sCJD) with combination of both expressive aphasia and NCSE.

Early recognition of potentially treatable etiologies can minimize the morbidity and mortality. Because of the delayed availability of results for autoimmune and paraneoplastic etiologies on CSF, we empirically used steroids to treat possible underlying autoimmune encephalopathy. Steroids can be empirically given in the setting of an elevated CSF protein (as our case) or personal or family history of an autoimmune disease [10]. However, there is not enough evidence to routinely recommend the use of steroids as a routine treatment in rapidly progressive dementia. We did not use intravenous immunoglobulin (IVIG) or plasmapheresis since we did not have enough evidence to support the diagnosis of AE [11].

CSF analysis for protein 14-3-3 has positive predictive value of 93% to 95% but its sensitivity is low [33]. Other CSF proteins such as tau and neuron-specific enolase are nonspecific general markers of neuronal injury and their utility is questionable due to lack of specificity [15].

The most common EEG finding in CJD is diffuse slowing pattern. However, the characteristic EEG findings for CJD are periodic synchronous bi- or triphasic or mixed sharp wave complexes (PSWCs) which have a specificity for sCJD ranging from 66% to 91% [1, 20, 34, 35]. However, its sensitivity is variable based on genotypes of sCJD [34]. PSWCs are sensitive to disease stage and external stimulation [20]. Lateralized PSWCs are seen during the disease course and represent the prodromal stage of disease onset, which then progresses in bifrontal distribution as disease advances as in our patient [35]. EEG-related spikes are independent of the traditional clinical finding of myoclonic jerking and are usually seen while patient is awake. Sleep deprivation usually exacerbate them, and benzodiazepines can mask the PSWCs [20]. We noticed similar pattern in our patient (Figures 1(c) and 1(d)).

Use of DWI or fluid attenuated inversion recovery (FLAIR) and apparent diffusion coefficient (ADC) modalities have significantly improved the sensitivity and specificity to 96% and 93%, respectively, of MRI for diagnosis of CJD [20, 26]. Hyperintensities in the putamen and head of the caudate nuclei are the most common findings on conventional MRI sequences in patients with CJD [36]. Other typical MRI findings in CJD include hyperintensities with diffusion restriction in the frontal, temporal, occipital, insular, and/or parietal regions referred to as cortical ribboning. Moreover, MRI is helpful to identify any other potential inflammatory, infectious, and toxic-metabolic causes of rapidly progressive dementia which might be mimicking CJD [20, 26]. Our patient had similar extensively worsening cortical ribboning pattern (Figure 2(d)).

MRI pattern may be affected by disease stage. DWI is considered superior during early stages of CJD [26]. Later during the disease course hyperintensity decreases and the only findings may be cortical atrophy [37]. Studies have shown that hyperintensity on DWI and ADC studies correlates with the symptoms and clinical course of the disease. Hyperintensity of basal ganglia on DWI is believed to be associated with a shorter disease duration and higher incidence of myoclonus [20]. Our patient had hyperintensity on basal ganglia and disease course was shorter and he had myoclonus (few weeks). One study reported a potential correlation between hyperintense lesions in the occipital cortex on DWI and shorter time between symptom onset and akinetic mutism [38]. Our patient did not show any occipital lobe lesions and did not develop akinetic mutism.

Definitive diagnosis can be established only with tissue diagnosis from brain biopsy or on autopsy tissue and genetic analysis is required as well. However, getting a biopsy is often not possible (as in our case) because of the concern for appropriate tissue handling and to prevent cross-contamination. Diagnostic yield of brain biopsy is low with traditional sampling methods [20] but newer techniques have shown better diagnostic yield [39]. We did not do biopsy as we have enough evidence from noninvasive work to substantiate the probable diagnosis of CJD. We recommend getting biopsy if it can potentially affect the treatment plan or disease course, and appropriate tissue handling is available.

Currently, there is no definite treatment available for this lethal condition. Antiepileptic drugs are used for myoclonic symptoms along with supportive and palliative care. There is no need to isolate the patient, but careful handling of CSF and brain tissue is strongly recommended.

Our patient had an atypical initial clinical presentation but with progressive changes in his radiological, EEG, and CSF findings becoming more typical and diagnostic for CJD. The progression of his imaging (MRI and EEG) and laboratory (CSF) abnormalities correlated well with his neurological deterioration. This emphasizes that uncommon etiologies must be considered in cases of unexplained and rapid neurological deterioration, especially with altered mentation and/or presence of refractory seizure activity. Close clinical monitoring is prudent and needs to be correlated with neuroimaging and cerebrospinal fluid analysis, which might aid in the early diagnosis of this lethal condition.

## 4. Conclusion

CJD can often have atypical clinical and radiological presentation. Diffuse epileptiform discharge (NCSE) on EEG in a patient with unexplained rapid cognitive decline and confusion might be a presentation of sCJD [14]. Potential reversible causes of rapidly progressive dementia such as autoimmune, infectious, and toxic-metabolic etiologies must be ruled out before making the final diagnosis of prion disease [12]. Continuous video EEG monitoring is crucial, especially if refractory epileptiform activity is suspected [40]. Due to the fatality of CJD, high degree of suspicion is prudent to initiate subsequent workup in instances of persistent/progressive unexplained neurocognitive decline and atypical clinical presentation and/or inconclusive initial workup.

## Figures and Tables

**Table 1:** Laboratory data.

Variable	Value
*Microbiology*	
CSF	Negative for bacterial growth
	Negative for acid fast bacilli (AFB)
*Autoimmune panel serum:*	
Microsomal TPO antibody	<0.2 IU/ml
Thyroxine binding globulin	19.3 microgram/ml
Anti-TG Ab	<0.2 IU/ml
HIV 1/2 PCR	None
Lyme	0.07 (Ref: ≤0.99 LIV)
FT Ab	Non-reactive
West Nile Virus (IgM)	None
SSA, 52 (Ro)	1 AU/ml
SSA, 60 (Ro)	1 AU/ml
Sjogren's Ab	0 AU/ml
ANA	None
Cysticercosis Ab, IgG by ELISA	0.0 (Ref: OD ≤ 0.34)
*Paraneoplastic antibodies serum:*	
ANNA (1–3)	Negative
AGNA-1	Negative
PCA (1-2)	<1 : 240 Negative
PCA-Tr	<1 : 240 Negative
Amphiphysin	<1 : 240 negative
CRMP-% IgG	<1 : 120 Negative
Striational Ab	0.0 nmol/L
P/Q type calcium channel Ab	0.0 nmol/L
	0.0 nmol/L
Ach receptor (muscle binding AB)	0.0 nmol/L
	0.0 nmol/L
Ach receptor (ganglionic neuronal Ab)	0.0 nmol/L
Neuronal (V-G) K+ channel Ab	0.0 nmol/L
*Other labs:*	
WBC	6.1 × 10^9^/L
Neutrophil	73%
Hemoglobin	15.9 g/dl
Hematocrit	45.3%
Platelets	211 × 10^9^/L
MCV	86 fl
Lymphocyte	30.70%
Eosinophil	1.88%
Polys	62%
Sodium	136 mEq/L
Potassium	3.5 mEq/L
Chloride	105 mEq/L
Bicarbonate	22 mEq/L
BUN	15 mg/dl
Creatinine	0.79 mg/dl
Anion Gap	9 mEq/L
Vitamin B 12	800 pg/ml
Thyroglobulin	2.0 ng/ml
TSH	4.016 microIU/ml
CRP	0.14 mg/L
AST	22 unit/L
ALT	21 unit/L
ALP	61 unit/L
Calcium	9.6 mg/dl
T-Bili	0.5 mg/dl
Albumin	4.4 g/dl
Total protein	7.5 g/dl

**Table 2:** Cerebrospinal fluid analysis.

CSF	Day 1	Day 12
*Number of tubes*	*2*	*4*
*Character/color*	*Colorless/clear*	*Colorless/clear*
*Volume *	*4 ml*	*20 ml*
*WBC *	*0 cells/unit*	*3 cells/unit*
*RBC *	*7 cells/unit*	*9 cells/unit*
*Lymphocytes*	*46%*	*14%*
*Mononuclear cells*	*54%*	*3%*
*Glucose CSF*	*63 mg/dl*	*66 mg/dl*
*Total protein, CSF*	*65 mg/dl*	*28 mg/dl*
*IgG CSF*	*3.5 mg/dl*	*-*
Lacrosse-California IgG		<1 : 1
Lacrosse-California IgM		<1 : 1
East Equine Virus IgG		<1 : 1
East Equine Virus IgM		<1 : 1
St Louis Virus IgG		<1 : 1
St Louis Virus IgM		<1 : 1
Western Equine Venezuela Virus IgG		<1 : 1
Western Equine Venezuela Virus IgM		<1 : 1
West Nile IgG, CSF		0.03
West Nile IgM, CSF		0.01
Varicella Zoster Virus		
Oligoclonal bands		
VDRL		Non-Reactive
Cocci Ab Ig G		0.1
Cocci Ab Ig M		0.0
*Coccidioides* AB ID		Not detected
Coccidioidomycosis Ab		<1 : 2
EBV, DNA quant interpretation		Not detected
EBV, Qnt log		<2.6 units: log
EBV, Quant source		CSF
EBV virus, Copy/m		<390 copy/ml
*Encephalitis/meningitis panel on CSF by PCR*		
E. *Coli K -1*		Not detected
H. *Influenza*		Not detected
L. *Monocytogenes*		Not detected
N *Meningitides*		Not detected
S. *agalactae*		Not detected
S. *Pyogenes*		Not detected
Cytomegalovirus		
Herpes Simplex Virus (HSV 1 & 2)		
Human herpes Virus-6		
Varicella Zoster Virus		
Cryptococcus Neoformans		
*Paraneoplastic Panel*		
AGNA-1 Amphiphysin Ab ANNA-1 Reflex Added ANNA-2 CRMP-5 PCA-1 and 2 PCA-Tr		Negative <1 : 2
*S-100B*		*3230 ng/L*
*RT QulC*		*POSITIVE*
*14-3-3 Protein*		*+++*
*T- Tau Protein*		*++ 9094 pg/ml*
